# Distributed Multi-Antenna Positioning for Automatic-Guided Vehicle

**DOI:** 10.3390/s20041155

**Published:** 2020-02-20

**Authors:** Xinyuan An, Sihao Zhao, Xiaowei Cui, Qin Shi, Mingquan Lu

**Affiliations:** 1 Department of Electronic Engineering, Tsinghua University, Beijing 100084, China; 2 Luoyang Electronic Equipment Test Centre, Luoyang 471000, China; 3 Beijing National Research Center for Information Science and Technology, Beijing 100084, China

**Keywords:** automatic-guided vehicles, distributed multi-antenna positioning, Levenberg–Marquardt

## Abstract

Radio-based positioning systems are typically utilized to provide high-precision position information for automatic-guided vehicles (AGVs). However, the presence of obstacles in harsh environments, as well as carried cargoes on the AGV, will degrade the localization performance, since they block the propagation of radio signals. In this paper, a distributed multi-antenna positioning system is proposed, where multiple synchronous antennas are equipped on corners of an AGV to improve the availability and accuracy of positioning. An estimator based on the Levenberg–Marquardt algorithm is introduced to solve the nonlinear pseudo-range equations. To obtain the global optimal solutions, we propose a coarse estimator that utilizes the displacement knowledge of the antennas to provide a rough initial guess. Simulation results show a better availability of our system compared with the single antenna positioning system. Decimeter accuracy can be obtained under a Gaussian measurement noise with a standard deviation of 0.2 m. The results also demonstrate that the proposed algorithm can achieve positioning accuracy close to the theoretical Cramer–Rao lower bound. Furthermore, given prior information of the yaw angle, the same level of accuracy can be obtained by the proposed algorithm without the coarse estimation step.

## 1. Introduction

Automatic-guided vehicles (AGVs) play an important role in unmanned ports or warehouses. They can automatically and safely transport cargoes or materials to the expected destination. Since the 1980s, AGV became an indispensable part of the production logistics system. In order to realize the safe driving of AGVs and the accurate lifting of cargoes in unmanned environments, autonomous high-precision positioning and navigation of AGV are necessary.

In the early days, AGVs mainly used fixed path methods for navigation [1]. In these methods, positioning signs such as magnetic stripes or metal wires are embedded in paths, and the sensors on the AGV can then detect the signs and provide information to the controller for driving adjustment. Although this method is mature and it was widely used in early AGVs, it only works for a fixed path and can be easily affected by the magnetic and electric fields in the environment. With the increasing demand for flexibility and adaptability of the AGV navigation system, positioning methods free of path constraints are constantly being researched and promoted, including inertial, radio, laser, and visual positioning, among others [2]. The inertial navigation system (INS) can provide high-frequency velocity and attitude estimation by using inertial sensors installed on the vehicle. It gives an ephemeral precise positioning by dead reckoning, immune to external interference. However, it suffers significant accumulated errors in the long run. The laser positioning system uses laser scanning to obtain the location information of reflection signs to provide vehicle position. It has high precision, but it is susceptible to weather and needs a large number of reflective signs [2]. The visual positioning system provides localization services by processing images collected by visual sensors. It achieves high positioning accuracy in various scenes, albeit with a high cost and computational complexity. Due to the development of radio technology and data processing abilities of electronic devices, the radio positioning system became favored in recent years, among which the global navigation satellite system (GNSS) and ultra-wideband (UWB) system are particularly concerned.

GNSS is a wireless navigation system that provides global, all-weather, continuous, and real-time navigation and positioning by utilizing navigation satellites [3]. Centimeter-level accuracy can be achieved by carrier phase differencing technology, which can meet the positioning accuracy requirements of AGVs. However, GNSS signals are subject to blockage and multipath, which decrease the continuity and availability of the carrier phase measurements and further lower the precision, robustness, and availability of positioning [4,5]. Moreover, it is not suitable for indoor positioning due to significant signal loss [6,7].

Recently, we saw a trend of utilizing UWB as a navigation system. UWB radios have relative bandwidths larger than 20% or absolute bandwidths of more than 500 MHz [8,9]. UWB signals are particularly well suited for localization since they can provide accurate and reliable ranging measurements due to their fine time resolution and robustness in harsh environments [10]. Typically, centimeter-level accuracy can be obtained for UWB by using time-of-arrival (TOA) measurements [11,12]. Furthermore, the UWB positioning system can be configured for indoor applications [13,14]. With the development of UWB chips, UWB became a popular technology for regional navigation [15]. Similar to GNSS, the UWB navigation system also encounters the problem of accuracy and availability degradation caused by signal blockage [16,17].

Aiming at the problem of insufficient measurements caused by obstacles in the environment, researchers proposed a variety of solutions. Integrating radio navigation systems with other sensors such as INS [18,19], velocity sensor, Light Detection and Ranging (LiDAR) [20,21], or visual sensor [22] represents a solution. This fusion method is helpful to improve the availability but needs additional external devices, and the data fusion of different systems requires much more computation ability. In addition to the multi-sensor fusion method, the idea of cooperative localization is another solution. It solves the problem of insufficient information of a single node by sharing information among nodes [23,24]. Specifically, all users are nodes operating independently with unknown locations and asynchronous clocks. These users make measurements with both known location references and other unknown location nodes. The additional information gained from these measurements between pairs of unknown location nodes enhances the accuracy and robustness of the localization system [25]. Some studies showed that the cooperative technology can effectively improve the availability of the positioning system in harsh environments, and it is suitable for location service applications with a group of agent nodes [26,27]. However, it strongly depends on wireless communication networks, which requires stable operation of networks and reliable transmission of information.

**Figure 1:**
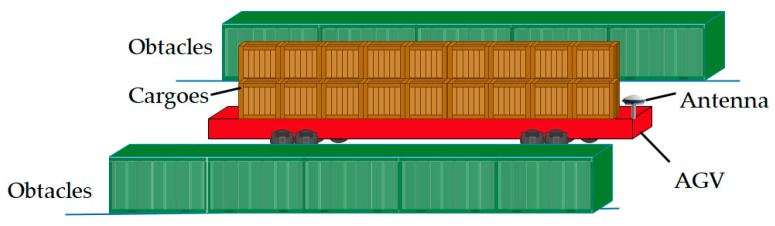
The flat structure of an automatic-guided vehicle (AGV) and its typical environment. The receiving antennas cannot be mounted at the highest point of the vehicle. The radio positioning signals are not only affected by the obstacles in the environment but also blocked by the carried cargoes.

In addition to all these difficulties mentioned above, another issue should be taken into account as shown in Figure 1. The red vehicle is an AGV, which has a flat structure. The cargoes are placed on the flat for transportation and there is no cab. Because of the restriction of the flat body, the receiving antennas cannot be mounted at the highest point of the vehicle. Thus, for the AGV, the radio positioning signals are not only affected by the obstacles in the environment but also blocked by the carried cargoes. The problem of insufficient measurements is even more serious in this case. Therefore, a method that can obtain as many measurements as possible and make full use of them is needed. Although more measurements will lead to an increase in computation, it is worth the improvement in availability, which is a prerequisite for the effective operation of the positioning system.

A multi-antenna method is our choice. Because of the spatial separation between antennas, more potential information can be provided for positioning from multiple antennas. Furthermore, redundant measurements by multiple antennas can improve the performance of positioning in terms of accuracy and robustness. Although the multi-antenna method was considered in many localization methods to improve localization [28,29,30,31], to the best of the authors’ knowledge, there are few studies on multi-antenna positioning systems in harsh environments. Some relevant studies typically only focused on multiple receivers. Some studies [32,33] improved the accuracy and reliability of positioning by using multiple low-cost receivers. These papers verified the accuracy improvement by utilizing spatially separated reception and data fusion, but did not discuss the problem of availability. Another study [33] set the position, attitude, velocity, angular rate of the center receiver, and clock bias of each receiver as unknown states and integrated the pseudo-range measurements from multiple global positioning system (GPS) receivers using an extended Kalman filter (EKF), which needs more measurements than single-receiver methods regardless of the availability. Multiple receivers were also used [34] to jointly track and estimate signal parameters to improve the reliability and robustness, but this study involved modification of the software and hardware of the tracking loop in the receivers.

Instead of multiple receivers, multiple distributed antennas with a synchronous clock are employed in this work. In our positioning system, fixed anchors periodically transmit positioning signals. Furthermore, there exist obstacles occasionally blocking the signals, such as buildings or containers on the roadside. The anchors can be automatically clock-synchronized and self-localized using a signal broadcast mechanism [35]. The distributed receiving antennas are mounted on the flat body of the AGV. The positions of these antennas are fixed and known with respect to a reference point on the vehicle. Due to the spatial differences, each antenna may encounter different blockages and, thus, have different subsets of visible anchors. Fusing the measurements of each antenna can improve the availability of the positioning system. Utilizing the position relationship between the receiving antennas and the reference point, the TOA measurements of each antenna can be expressed as a function of the reference position. In order to implement the transformation of the measurement representation, a rotation matrix from the body coordinate system (frame *b*) to the navigation coordinate system (frame *n*) is inevitably needed. Therefore, unlike the single antenna positioning method, in our distributed multi-antenna (DMA) method, not only the position and clock bias but also the attitude of AGV should be estimated, which forms a challenging nonlinear constrained optimization problem. The problem of finding the rotation matrix and the reference position was termed rigid body localization in some studies [36,37,38]. They localized a rigid body by placing a few sensors on the body and utilized the range measurements with respect to a few anchors to estimate the rotation matrix and the position [36]. A non-iterative estimator with two steps was also proposed [37]. The rotation matrix and position were coarsely estimated using sensor positions and then refined by estimating the corrections using Euler angle formulation. Another study [36] proposed two methods called constrained least squares (CLS) and simplified CLS based on linearizing the measurement model. In Reference [38], this problem of maximum likelihood (ML) formulation was addressed by using semidefinite relaxation to get a coarse estimate, before applying orthogonalization and refinement. Although these methods showed good performance in their circumstances, they are not suitable for our cases because they ignore the problem of insufficient measurements from a single sensor, and they simply assume that there is no clock bias, which is not practical in many real applications. If the rigid body localization methods are directly applied to our scene, the TOA measurements are treated as range measurements with large noise and the number of measurements is small, leading to low accuracy.

In this paper, we propose a DMA positioning algorithm based on the Levenberg–Marquardt (LM) algorithm with a coarse estimation step, which is introduced to provide a proper initial value to the iterative positioning algorithm. In the coarse estimation, the positions of the distributed antennas in frame *n* are jointly estimated and then used to coarsely estimate the reference position and the yaw angle according to the transformation relationship between frame *b* and frame *n*. Finally, the coarse estimates are applied to the iterative algorithm to get a refined positioning result. Thus, the algorithm is divided into two steps, and the positioning algorithm based on LM is hereinafter referred to as the two-step LM (TSLM) positioning algorithm. The Cramer–Rao lower bound (CRLB) of the positioning problem is derived as a benchmark to assess the performance of the proposed algorithm. The distributed multi-antenna horizontal dilution of precision (DMA-HDOP) model is also derived to analyze the positioning accuracy. Simulation results demonstrate that the positioning availability is significantly improved compared with the conventional single-antenna localization. Under a Gaussian measurement noise with a standard deviation of 0.2 m, the DMA method could achieve decimeter-level accuracy. Furthermore, in our simulation tests, the TSLM algorithm could provide positioning accuracy close to CRLB without requiring the initial yaw angle information. Simulation tests on the second step of the TSLM algorithm with different initial yaw angles verified that the iterative positioning algorithm in the second step is sensitive to the initial yaw angle. However, given prior information of the yaw angle, using only the second step can achieve similar results to TSLM.

The main contributions of this paper are summarized as follows:

1) A distributed multi-antenna method is proposed to improve the availability and accuracy of AGV positioning under the situation of signal blockage caused by environmental obstacles and carried cargoes.

2) A two-step LM algorithm is developed to optimally estimate both the position and the yaw angle, in which the initial value for the iterative algorithm in the second step is set by the coarse estimation from the first step.

The remainder of the article is organized as follows: Section 2 presents the model of the positioning system. The positioning algorithms are proposed in Section 3. Section 4 derives the CRLB of the position estimate problem and presents the DMA-HDOP model of the DMA positioning method. Section 5 gives the simulation results. Finally, Section 6 concludes the paper.

## 2. System Model

### 2.1. Notation

We employ the following notations throughout this work: the upper and lower case boldface denote matrices and vectors, respectively; symbols with a hat denote estimated values; the notation E[·] is the expectation operator; $A^{T}$, $A^{-1}$, and trace(A) denote the transpose, the inverse, the trace of matrix A, respectively; ‖·‖ denotes the Euclidean norm; I is the identity matrix; card(ℬ) denotes the number of elements in set ℬ; C(n,k) denotes the number of *k*-combinations of *n* elements; $A_{n_{1}\times n_{2}}$ denotes the $n_{1}$-by-$n_{2}$ matrix; $a\left[n_{1}:n_{2}\right]$ denotes the $n_{1}$-th to $n_{2}$-th elements of vector a; a[n] denotes the n-th element of vector a; ${\left[A\right]}_{n_{1},n_{2}}$ denotes the element at the $n_{1}$-th row and $n_{2}$-th column of matrix A.

Coordinate frames involved in this paper are the navigation frame and the body frame, denoted as *n* and *-b*, respectively. All the positions of anchors and the positioning results are expressed in frame *n*, which defines a certain point in the given area as its origin and the east, north, and upward directions as its $x^{n}$, $y^{n}$, and $z^{n}$ axes, respectively. The known positions of the distributed antennas with respect to a reference point on AGV are expressed in frame *b*. The origin of frame *b* is the reference point. $x^{b}$, $y^{b}$, and $z^{b}$ axes of frame *b* are defined as the front, right, and downward directions of the AGV body, respectively. The rotation matrix of transformation from frame *b* to frame *n* is represented by $R_{b}^{n}$.

### 2.2. Problem Formulation

**Figure 2:**
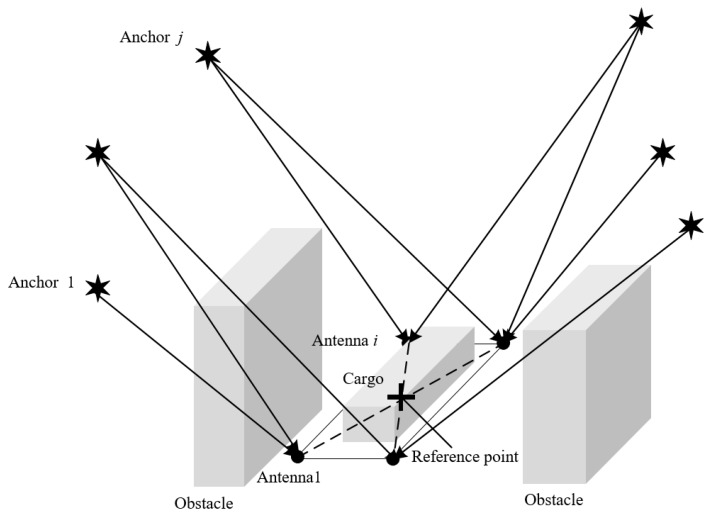
An example of a signal-blocked environment of AGV. The AGV is carrying some cargo, and the receiving antennas are installed on the corners of its flat body.

Consider a system in which there are M anchors with known position and known clock bias, an AGV with unknown position, and N antennas with unknown positions and the same unknown clock bias. Let N be the set of antennas with card(N)=N, ℳ be the set of anchors with card(ℳ)=M, and ${ℳ}_{i}$ be the set of visible anchors of antenna *i* with $\mathrm{card}\left({ℳ}_{i}\right)=M_{i}$, ${ℳ}_{i}\subset ℳ$. Position states of the reference point c on the AGV, antenna i∈N, and anchor j∈ℳ in frame *n* are indicated by $p_{c}=\left(x_{c},y_{c},z_{c}\right)$, $p_{i}=\left(x_{i},y_{i},z_{i}\right)$, and $p^{\left(j\right)}=\left(x^{\left(j\right)},y^{\left(j\right)},z^{\left(j\right)}\right)$, respectively. A typical signal blocked environment of the AGV using a radio positioning system is presented in Figure 2. The AGV is carrying some cargo, and the receiving antennas are installed on its flat body.

In unmanned transportation, the AGV is typically assumed to work in a flat and specific area without changing the height, pitch angle, and rolling angle. Therefore, the height, rolling angle, and pitch angle are regarded as known constants, and the problem can be considered as positioning in a two-dimensional (2D) plane. In addition to the horizontal coordinates of the reference point $\left(x_{c},y_{c}\right)$, the clock bias δt between the receiver and the anchor should also be estimated. Moreover, the yaw angle ψ is needed for the position vector transformation from frame *b* to frame *n*.

Three-dimensional (3D) AGV positioning is also needed in some application scenarios such as transportation on rough roads, open-air mines, etc., in which AGVs are moving with varying height and/or rolling and pitch angle. For the height estimate, a good geometry of anchor height should be additionally guaranteed. The height can then be solved by adding an extra unknown with an increase in the number of visible anchors required. As for the rolling and pitch angles, it is not only a matter of increasing the unknowns and the number of visible anchors required. The coupling between multiple attitude angles also brings more severe nonlinearity, which may require more sophisticated optimization algorithms, which will be investigated in future work. Fortunately, unlike the yaw angle, which is not easy to be measured directly by sensors, the rolling and pitch angles are easy to be accurately obtained using accelerometers and other sensors; thus, it is easy to extend the proposed method to 3D cases.

Based on the previous discussion, in this work, we focus on the 2D problem, and the parameters to be estimated are the reference position $\left(x_{c},y_{c}\right)$, receiver clock bias δt, and yaw angle ψ. The unknown state vector x can be written as

(1) $$x={\left[x_{c},y_{c},\delta t,\psi \right]}^{T}.$$

### 2.3. Measurement Model

In a broadcast positioning system, the receiver receives the signals transmitted from the anchors and measures the time of arrival with its local clock, which is not synchronized with the anchors. Assuming that all the anchors are synchronized to a system clock, the TOA measurement of the receiving antenna *i* from anchor *j* can be formulated as

(2) $$\rho_{ij}=\left\|p^{\left(j\right)}-p_{i}\right\|+\delta t_{i}+\varepsilon_{ij},$$

where $\delta t_{i}$ is the receiver clock bias expressed by the equivalent signal propagation distance, and $\varepsilon_{ij}$ is the TOA measurement noise. Note that the measurement noises come from various sources, including the geometry of anchors, multipath effect, clock inaccuracies, etc. They are random variables, and it is very difficult to get their exact probability distribution. Without loss of generality, all the measurements noises are assumed to be independent and identically distributed Gaussian white noise, i.e., $\varepsilon_{ij}∼N\left(0,\sigma^{2}\right)$.

**Figure 3:**
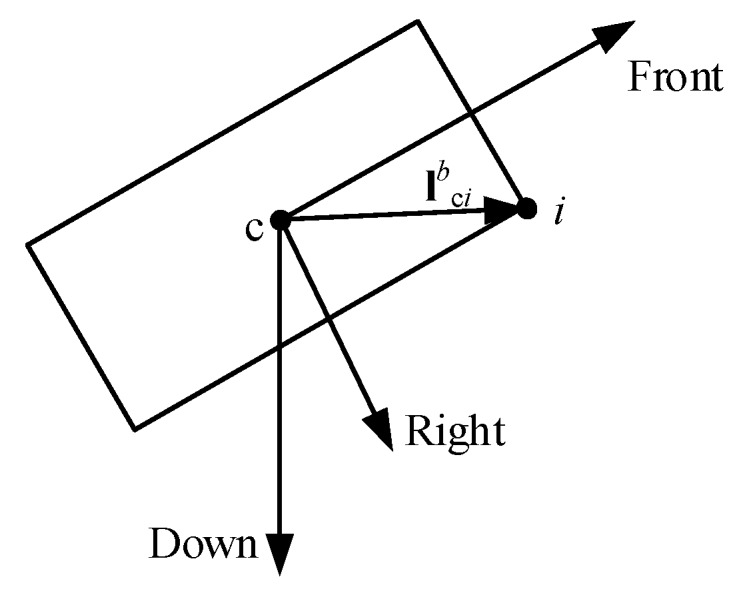
The position of antenna *i* in frame *b*; c is the reference point on the AGV.

For our distributed multi-antenna positioning system, the position relationship between the antennas and the reference point is shown in Figure 3. For the distributed antennas, their positions relative to the reference point c are known and fixed in frame *b*, whereas the orientation of frame *b* varies with the motion of the AGV. The position of antenna *i* in frame *n* can be calculated as

(3) $$p_{i}=p_{c}+R_{b}^{n}l_{ci}^{b},\;i\in N,$$

where $l_{ci}^{b}$ is the vector from the reference point c to antenna *i* expressed in frame *b*, and $R_{b}^{n}$ is the rotation matrix from frame *b* to frame *n* [18].

(4) $$R_{b}^{n}=\left[\begin{array}{ccc} \sin\psi \cos\theta & \cos\psi \cos\phi +\sin\psi \sin\theta \sin\phi & -\cos\psi \sin\phi +\sin\psi \sin\theta \cos\phi \\ \cos\psi \cos\theta & -\sin\psi \cos\phi +\cos\psi \sin\theta \sin\phi & \sin\psi \sin\phi +\cos\psi \sin\theta \cos\phi \\ \sin\theta & -\cos\theta \sin\phi & -\cos\theta \cos\phi \end{array}\right],$$

where the rolling angle ϕ, pitch angle θ, and yaw angle ψ are defined as in Reference [18]. $R_{b}^{n}$ is a function of yaw angle ψ in this 2D positioning case.

Substituting the expression of $R_{b}^{n}$ into Equation (3), the position of antenna *i* is a function of the reference position $\left(x_{c},y_{c}\right)$ and the yaw angle ψ, as given by

(5) $$p_{i}=f_{i}(x_{c},y_{c},\psi ).$$

With the same timing source, all antennas have the same clock bias δt. Substituting Equation (5) into Equation (2), the TOA measurement $\rho_{ij}$ of the *j*-th anchor in the subset of visible anchors ${ℳ}_{i}$ of antenna *i* is

(6) $$\rho_{ij}=\left\|p^{\left(j\right)}-f_{i}(x_{c},y_{c},\psi )\right\|+\delta t+\varepsilon_{ij},\;i\in N,\;j\in {ℳ}_{i}.$$

The unknowns in Equation (6) are $x_{c}$, $y_{c}$,  δt, and ψ. Unlike the single-antenna positioning equation of Equation (2), the yaw angle ψ is added.

For convenience, Equation (6) is rewritten into matrix form,

(7) z=h(x),

where h(x) is the observation function. The observation vector z consists of the TOA measurements of all N antennas given by Equation (6). The total number of measurements is $\sum_{i=1}^{N}M_{i}$. For antenna *i*, the TOA measurement $\rho_{i}$ is

(8) $$\rho_{i}={\left[\begin{array}{cccc} \rho_{i1} & \rho_{i2} & \cdots & \rho_{iM_{i}} \end{array}\right]}^{T}.$$

h(x) consists of the TOA measurement function of all N antennas. For antenna *i*, the TOA measurement function $h_{i}(x)$ is

(9) $$h_{i}(x)={\left[\begin{array}{cccc} h_{i1}(x) & h_{i2}(x) & \cdots & h_{iM_{i}}(x) \end{array}\right]}^{T},$$

where $h_{ij}\left(x\right)$ is expressed as the right side of Equation (6).

The aim of the localization problem in this paper is to estimate the position, orientation, and clock bias parameters for an AGV. Utilizing the measurements given by Equation (7), the positioning algorithm is proposed in the next section.

## 3. Positioning Algorithm

To estimate the unknowns in a set of equations, the number of independent equations must be greater than or equal to the number of unknowns in the system. Therefore, the corresponding requirement is that the total number of visible anchors for all the distributed antennas must be greater than or equal to the dimension of x in Equation (1). In addition, to estimate the yaw angle, there should be more than two antennas, where each observes at least one anchor. Let $X=\left\{M_{i}>0,i\in N\right\}$. If

(10) $$\sum_{i=1}^{N}M_{i}\geq 4\;\mathrm{and}\;\mathrm{card}\left(X\right)\geq 2,$$

the unknown parameters in x can be solved. We note that, for the positioning case with a single antenna, the number of visible anchors should be at least three to determine the two-axis position and the clock bias terms. The condition in Equation (10) shows that this requirement is relaxed in the multiple-antenna case, i.e., the number of visible anchors for each antenna can be less than three. As long as it is satisfied that the total number of visible anchors is greater than four, and no fewer than two antennas can see one or more anchors, the position of the target can be obtained. This greatly improves the availability of the system in practice.

According to the above models, the problem of AGV positioning is to find an optimal estimate $\hat{x}$ with the minimum variance. The cost function is

(11) $$J(x)=\sum_{i=1}^{N}\sum_{j=1}^{M_{i}}{\left(\rho_{ij}-\left\|p^{\left(j\right)}-f_{i}(x_{c},y_{c},\psi )\right\|-\delta t\right)}^{2}.$$

Note that, because the TOA measurement noise of each antenna is assumed to be independent and identically distributed, the weights used to represent the contributions of the measurements are then chosen to be in unity and, thus, are omitted in the cost function. The parameters are estimated by solving the following minimization problem:

(12) $$\hat{x}=\underset{x}{\arg\min}\;J(x).$$

The optimization problem in Equation (12) can be converted to a nonlinear least squares problem and solved by an iterative method such as the Newton iterative method [39].

However, the parameters to be estimated in this problem show high nonlinearities in the measurement equations. Therefore, to avoid a local minimum solution of the optimization problem, a reasonably good starting point is needed. For the time parameter, it is a linear item in the measurement model which can be set empirically or to zero for simplicity. For the space parameters, the initial position can be set according to the region formed by the anchors, e.g., the center of the region. In contrast, the yaw angle, which is expressed as a trigonometric function in the equations, has the strongest nonlinearity among all these parameters, and its initial value has great influence on the convergence of the optimization problem. This impact on convergence is demonstrated later in the simulations in Section 5.2. If there are some other sensors such as an INS that can provide the attitude information of the body, the iterative positioning algorithm can be applied directly. However, more generally, we have no prior information about the yaw angle, and the AGV may start at the same location but with completely different yaw angles; thus, its initial value may be a randomly guessed value from −π to π rad. Therefore, some measures are needed to narrow the initial attitude guess within a proper accuracy to guarantee convergence to the global optimum.

**Figure 4:**
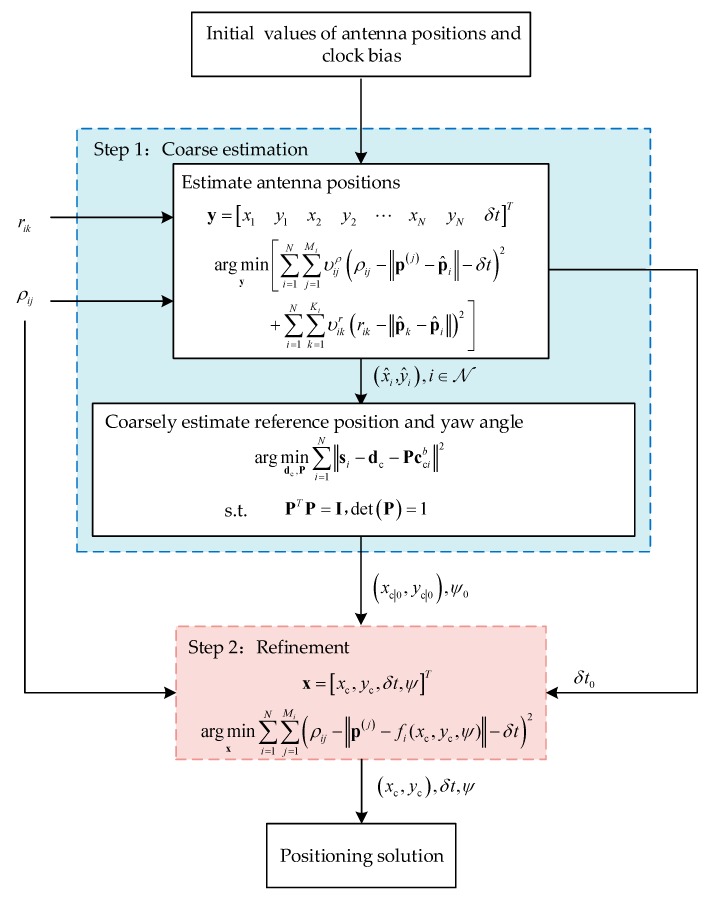
Flow chart of the two-step Levenberg–Marquardt (TSLM) algorithm.

Toward that end, we employ a coarse estimation process to the algorithm. In this process, the antenna positions are jointly estimated as intermediate variables with the initial values of space and time parameters as described in the last paragraph, which avoids randomly guessing the initial yaw angle. Moreover, the initial reference position $\left(x_{c|0},y_{c|0}\right)$ and the yaw angle $\psi_{c|0}$ are then calculated according to the transformation relationship of antenna positions between frame *b* and frame *n*. Finally, the coarse estimates of space, time, and attitude serve as initial values in the iterative positioning algorithm. Consequently, a refined estimate is obtained. With the coarse estimation process, the DMA positioning algorithm based on the LM algorithm can be divided into two steps, namely, the TSLM algorithm, as shown in Figure 4, which is presented in detail in the upcoming subsections.

### 3.1. Step 1: Coarse Estimation

In Step 1, coarsely estimated parameters are obtained by firstly estimating antenna positions and then determining the reference position and the rotation matrix utilizing the relationship between frame *n* and frame *b*.

#### 3.1.1. Estimate Antenna Positions

According to the TOA measurements expressed in Equation (2), the position of antenna *i* or $p_{i}$ can be estimated if there are sufficient independent measurements between antenna *i* and its visible anchors ${ℳ}_{i}$. As mentioned before, due to the obstacles both in the environment and on the AGV, the number of TOA measurements of a single antenna may not be enough for positioning. Thus, we adopt the idea of collaborative positioning in Reference [40], where a group of receivers with unknown locations and asynchronous clocks were localized by jointly using the TOA measurements of all these receivers and the distance measurements between them. What distinguishes our work from the existing collaborative localization system is that the distance measurements between antennas are calculated according to their known positions in frame *b* and the antennas share a synchronous clock. The parameters to be estimated are positions of the antennas and receiver clock bias δt. The unknown state vector y can be written as

(13) $$y={\left[\begin{array}{ccccc} \begin{array}{cc} x_{1} & y_{1} \end{array} & \begin{array}{cc} x_{2} & y_{2} \end{array} & \cdots & \begin{array}{cc} x_{N} & y_{N} \end{array} & \delta t \end{array}\right]}^{T}.$$

We can rewrite the TOA measurement $\rho_{ij}$ of the *j*-th anchor in the subset of visible anchors ${ℳ}_{i}$ of antenna *i* as

(14) $$\rho_{ij}=\left\|p^{\left(j\right)}-p_{i}\right\|+\delta t+\varepsilon_{ij},\;j\in {ℳ}_{i},$$

where $\varepsilon_{ij}∼N\left(0,\sigma^{2}\right)$.

The distance measurement between antenna *i* and antenna *k* can be expressed as

(15) $${\hat{r}}_{ik}=\left\|{\hat{p}}_{k}-{\hat{p}}_{i}\right\|.$$

The true distance is calculated by

(16) $$r_{ik}=\left\|l_{ck}^{b}-l_{ci}^{b}\right\|,$$

with the known positions of the distributed antennas in frame *b* $l_{ci}^{b}$, i∈N.

Although $r_{ik}$ and $r_{ki}$ are expressed as two measurements, they should be counted as one. For N antennas, there are C(N,2) independent distance measurements. Let $K_{i}$ be the set of antennas having effective distance measurements with antenna *i*, $\mathrm{card}\left(K_{i}\right)=K_{i}$, $K_{i}\subset N$.

If the sum of the number of the TOA measurements and distance measurements is greater than or equal to the dimension of y, y can be solved. Thus, the requirement is

(17) $$\sum_{i=1}^{N}M_{i}+C\left(N,2\right)\geq 2N+1,$$

which can be easily met by reasonably deploying anchors, especially at the starting point of the vehicle. For example, there are four antennas mounted on the AGV and, thus, six distance measurements. According to Equation (17), the number of TOA measurements should be no less than three. 

Jointly utilizing the TOA measurements and the distance measurements, an optimal estimate $\hat{y}$ can be obtained by

(18) $$\hat{y}=\underset{y}{\arg\min}\left[\sum_{i=1}^{N}\sum_{j=1}^{M_{i}}\upsilon_{ij}^{\rho }{\left(\rho_{ij}-\left\|p^{\left(j\right)}-{\hat{p}}_{i}\right\|-\delta t\right)}^{2}+\sum_{i=1}^{N}\sum_{k=1}^{K_{i}}\upsilon_{ik}^{r}{\left(r_{ik}-\left\|{\hat{p}}_{k}-{\hat{p}}_{i}\right\|\right)}^{2}\right].$$

where $\upsilon_{ij}^{\rho }$ and $\upsilon_{ik}^{r}$ are the weights for the TOA measurements and the distance measurements, respectively. According to the assumptions for TOA measurement noises, $\upsilon_{ij}^{\rho }$ can be chosen as $\upsilon_{ij}^{\rho }=\sigma^{-2}$. The distance measurements should have heavier weights due to their high accuracy. Here, we set $\upsilon_{ik}^{r}=\alpha \sigma^{-2}$, where α is a factor greater than 1.

By linearizing the measurement equations by Taylor expanding and keeping the first-order terms, we have

(19) δq=Kδy,

where $\delta y=y-\hat{y}$.

(20) $$K=\left[\begin{array}{c} K_{\rho }\\ K_{r} \end{array}\right],\;\delta q=\left[\begin{array}{c} \delta q_{\rho }\\ \delta q_{r} \end{array}\right].$$

The parameters related to the TOA measurements are denoted with subscript ρ.

(21) $$K_{\rho }=\left[\begin{array}{ccccc} K_{\rho }{}_{,1} & & & & 1\\ & K_{\rho }{}_{,2} & & & 1\\ & & \ddots & & \vdots \\ & & & K_{\rho }{}_{,N} & 1 \end{array}\right],\;\delta q_{\rho }=\left[\begin{array}{c} \delta q_{\rho }{}_{,1}\\ \delta q_{\rho }{}_{,2}\\ \vdots \\ \delta q_{\rho }{}_{,N} \end{array}\right],$$

where

(22) $$K_{\rho ,i}=\left[\begin{array}{c} -u_{i1}\\ -u_{i2}\\ \vdots \\ -u_{iM_{i}} \end{array}\right],\;\delta q_{\rho ,i}=\left[\begin{array}{c} \Delta \rho_{i1}\\ \Delta \rho_{i2}\\ \vdots \\ \Delta \rho_{iM_{i}} \end{array}\right],$$

where $\Delta \rho_{ij}=\rho_{ij}-\left\|p^{\left(j\right)}-{\hat{p}}_{i}\right\|-\delta \hat{t}$, and

(23) $$u_{ij}≜\left[\frac{x^{\left(j\right)}-x_{i}}{d_{ij}},\frac{y^{\left(j\right)}-y_{i}}{d_{ij}}\right],$$

where $d_{ij}=\left\|p^{\left(j\right)}-f_{i}(x_{c},y_{c},\psi )\right\|$.

The parameters related to the distance measurements are denoted with subscript r

(24) $$K_{r}=\left[\begin{array}{c} K_{r}{}_{,1}\\ K_{r}{}_{,2}\\ \vdots \\ K_{r}{}_{,N} \end{array}\right],\;\delta q_{r}=\left[\begin{array}{c} \delta q_{r}{}_{,1}\\ \delta q_{r}{}_{,2}\\ \vdots \\ \delta q_{r}{}_{,N} \end{array}\right],$$

where

(25) $$K_{r,i}=k\text{-}\mathrm{th}\mathrm{row}\left[\begin{array}{cccccc} \vdots & \vdots & \vdots & \vdots & \vdots & \vdots \\ \overset{i-1}{\overset{︷}{\begin{array}{cc} 0_{1\times 2} & \cdots \end{array}}} & v_{ik} & \overset{index-i-1}{\overset{︷}{\begin{array}{cc} 0_{1\times 2} & \cdots \end{array}}} & -v_{ik} & \overset{N-index}{\overset{︷}{\begin{array}{cc} 0_{1\times 2} & \cdots \end{array}}} & 0\\ \vdots & \vdots & \vdots & \vdots & \vdots & \vdots \end{array}\right],\;\delta q_{r,i}=\left[\begin{array}{c} \Delta r_{i1}\\ \Delta r_{i2}\\ \vdots \\ \Delta r_{iK_{i}} \end{array}\right],$$

where *index* denotes the index of the *k*-th element of set $K_{i}$ in set N and is supposed to be greater than *i*; $\Delta r_{ik}=r_{ik}-\left\|{\hat{p}}_{k}-{\hat{p}}_{i}\right\|$, and

(26) $$v_{ik}≜\left[\frac{x_{k}-x_{i}}{r_{ik}},\frac{y_{k}-y_{i}}{r_{ik}}\right],\;k\in K_{i}.$$

Thus, the linear least square form of the cost function is

(27) $$J\left(\delta y\right)={\left(\delta q-K\delta y\right)}^{T}W\left(\delta q-K\delta y\right),$$

where W is the weighting matrix.

(28) $$W=\left[\begin{array}{cc} \sigma^{-2}I_{\rho } & \\ & \alpha \sigma^{-2}I_{r} \end{array}\right],$$

where $I_{\rho }$ and $I_{r}$ are $\sum_{i=1}^{N}M_{i}$-order and C(N,2)-order identity matrices, respectively. The solution to estimate δy is given by

(29) $$\delta \hat{y}=\arg\underset{\delta y}{\min}\;J(\delta y)={\left(K^{T}WK\right)}^{-1}K^{T}W\delta q.$$

The estimate of y can then be obtained by using the iterative algorithm. Consequently, the estimated $\left({\hat{x}}_{i},{\hat{y}}_{i}\right)$ and $\delta \hat{t}$ are obtained and utilized in the next steps.

#### 3.1.2. Coarsely Estimate Reference Position and Yaw Angle

In this subsection, we impose Equation (3) to determine the rotation matrix and the reference position, which is a typical problem in pattern analysis [37,41,42].

For our 2D problem, we denote the 2D positions of antenna *i* in frame *b* and frame *n* as $c_{i}^{b}$ and $s_{i}$, respectively, where $c_{i}^{b}=l_{ci}^{b}\left[1:2\right]$ and $s_{i}={\left[\begin{array}{cc} x_{i} & y_{i} \end{array}\right]}^{T}$. We can denote the 2D position of the reference point in frame *n* as $d_{c}={\left[\begin{array}{cc} x_{c} & y_{c} \end{array}\right]}^{T}$ and the rotation matrix between the two frames as

(30) $$P=\left[\begin{array}{cc} \sin\psi & -\cos\psi \\ \cos\psi & \sin\psi \end{array}\right].$$

Then, Equation (3) can be rewritten as

(31) $$s_{i}=d_{c}+Pc_{ci}^{b}.$$

We construct the cost function as

(32) $$J\left(d_{c},P\right)=\sum_{i=1}^{N}{\left\|s_{i}-d_{c}-Pc_{ci}^{b}\right\|}^{2}.$$

We assume that each estimated antenna position has a similar contribution to the cost function, and the weights are then chosen to be in unity and are omitted in the cost function. The problem can then be formulated as a least squares minimization problem,

(33) $$\arg\underset{d_{c},P}{\min}\;J=\arg\underset{d_{c},P}{\min}\sum_{i=1}^{N}{\left\|s_{i}-d_{c}-Pc_{ci}^{b}\right\|}^{2},\;s.t.\;P^{T}P=I,\;\det\left(P\right)=1.$$

We can utilize the method introduced in Reference [37],

(34) $$d_{c}=\bar{s}-P\bar{c},$$

where $\bar{s}=\frac{1}{N}\sum_{i=1}^{N}s_{i}$, $\bar{c}=\frac{1}{N}\sum_{i=1}^{N}c_{ci}^{b}$.

The minimization problem in Equation (33) can then be equivalent to the following [37]:

(35) $$\arg\underset{P}{\max}\mathrm{trace}\left(P\sum_{i=1}^{N}{\tilde{c}}_{ci}^{b}{\tilde{s}}_{i}^{T}\right),$$

where ${\tilde{s}}_{i}=s_{i}-\bar{s}$, ${\tilde{c}}_{ci}^{b}=c_{ci}^{b}-\bar{c}$.

Let

(36) $$U\Sigma V^{T}=\mathrm{SVD}\left(\sum_{i=1}^{N}{\tilde{c}}_{ci}^{b}{\tilde{s}}_{i}^{T}\right).$$

By referring to the method based on singular value decomposition (SVD) in Reference [37],

(37) $$P=V\left[\begin{array}{cc} 1 & 0\\ 0 & \det{\left(VU^{T}\right)}^{T} \end{array}\right]U^{T}.$$

Then,

(38) $$\psi =\arctan\left(\frac{{\left[P\right]}_{1,1}}{{\left[P\right]}_{1,2}}\right).$$

Upon substituting P into Equation (34), $d_{c}$ can be solved.

At this point, the coarsely estimated reference position and the yaw angle are all obtained. Although there are some errors in the coarse estimates due to the intermediate transformation, they are not far from the optimum. Simulations validated that their accuracy is sufficient to initiate the iteration in Step 2 for producing a refined solution achieving CRLB.

### 3.2. Step 2: Refinement

To get the refined solutions, the coarse estimates denoted as $\left(x_{c|0},y_{c|0}\right)$, $\delta t_{0}$, and $\psi_{0}$ are applied to Equation (12).

Firstly, the model of Equation (7) is linearized. Let $\hat{x}$ be the estimated values; then, the first-order Taylor expansion of h(x) at $\hat{x}$ is

(39) $$h(x)=h(\hat{x})+{\frac{\partial h}{\partial x}|}_{\hat{x}}(x-\hat{x})+o(x-\hat{x}),$$

where $o(x-\hat{x})$ is the high-order residual, which is ignored in the following text.

Let $\delta z=h\left(x\right)-h\left(\hat{x}\right)$, $\delta x=x-\hat{x}$, and $H={\frac{\partial h}{\partial x}|}_{\hat{x}}$; then, we have

(40) δz=Hδx.

Define

(41) $$w_{ij}≜\frac{\partial h_{ij}(x)}{\partial \psi },$$

and we have

(42) $$H=\left[\begin{array}{c} H_{1}\\ H_{2}\\ \vdots \\ H_{N} \end{array}\right],\;\delta z=\left[\begin{array}{c} \delta z_{1}\\ \delta z_{2}\\ \vdots \\ \delta z_{N} \end{array}\right],$$

where

(43) $$H_{i}=\left[\begin{array}{ccc} \begin{array}{c} -u_{i1}\\ -u_{i2}\\ \vdots \\ -u_{iM_{i}} \end{array} & \begin{array}{c} 1\\ 1\\ \vdots \\ 1 \end{array} & \begin{array}{c} w_{i1}\\ w_{i2}\\ \vdots \\ w_{i}{}_{M_{i}} \end{array} \end{array}\right],\;\delta z_{i}=\left[\begin{array}{c} \rho_{i1}-{\hat{\rho }}_{i1}\\ \rho_{i2}-{\hat{\rho }}_{i2}\\ \vdots \\ \rho_{iM_{i}}-{\hat{\rho }}_{iM_{i}} \end{array}\right],$$

in which,${\hat{\rho }}_{ij}=h_{ij}\left(\hat{x}\right)$.

Thus, the linear least square form of Equation (11) is

(44) $$J\left(\delta x\right)={\left(\delta z-H\delta x\right)}^{T}\left(\delta z-H\delta x\right).$$

The solution to estimate δx is given by

(45) $$\delta \hat{x}=\arg\underset{\delta x}{\min}\;J(\delta x)={\left(H^{T}H\right)}^{-1}H^{T}\delta z.$$

The estimate of x can then be obtained using the iterative algorithm.

During iteration, matrix H in Equation (45) must be full rank, or the condition number of matrix $Q=H^{T}H$ cannot be too high to ensure convergence of the algorithm. To this end, we refer to the LM algorithm. The LM algorithm is a trust region method that improves the condition number of matrix Q by increasing coefficient matrix damping [43].

In the LM algorithm, a positive damping coefficient μ is added to the matrix Q, that is,

(46) $$Q^{\prime }=H^{T}H+\mu I.$$

$Q^{\prime }$ is used to calculate the increment of the unknown parameters. It can be seen that, if μ=0, it is the same as Q. The damping coefficient should be adjusted after each iteration. Since μ is positive, the matrix $Q^{\prime }$ is always positive definite. In the iterative process, an error index is obtained by comparing the results of the current and the last iteration, which determines how to further update the damping coefficient. For a given μ, the commonly used adjustment strategy is as follows: if the residual result can reduce the error index, continue to reduce μ; otherwise, increase it. The details of the iterative positioning algorithm are shown in Algorithm 1.

**Algorithm 1.** Iterative positioning algorithm in Step 2**Input:** TOA measurements ***ρ**_i_*, i∈N; anchor positions $p^{\left(j\right)}$, $j\in {ℳ}_{i},\;{ℳ}_{i}⸦ℳ$; initial unknowns $x_{0}\;=\;{\left[x_{c|0},\;y_{c|0},\;y_{c|0},\;\delta t_{0},\;\psi_{0}\right]}^{T}$; maximum iterative number *iter*; convergence threshold *thr* > 0; known height *h*, rolling angle *φ*, pitch angle *θ* and the vectors from the antenna *i* to reference point c expressed in frame *b*
$l_{ci}^{b},\;i\in N$; damping coefficient *μ* > 0; adjustment coefficient *λ* > 1.
**Iteration:**
  for *q* = 1: *iter*    Calculate the position of each antenna:    ${\hat{p}}_{i}=f_{i}(x_{c|q-1},\;y_{c|q-1},\;\psi_{q-1}),\;i\in N.$    Calculate residual: $\delta \rho_{ij}=\rho_{ij}-\left\|p^{\left(j\right)}-{\hat{p}}_{i}\right\|-\delta t_{q-1},\;i\in N,\;,j\in {ℳ}_{i},\;{ℳ}_{i}⸦ℳ.$    Calculate **u**_*ij*_ and *w_ij_* according to (23) and (41), respectively.    Form **H** and *δ***z** according to (42).    Calculate **Q^′^** according to (46).    Calculate unknown incremental: $\delta x_{q}\;={Q^{\prime }}^{-1}H^{T}\delta z.$    Update unknown estimate: $x_{q}=x_{q-1}+\delta x_{q}.$     if ‖*δ***x**_*q*_‖ < *thr*      exit;     else if ‖*δ***x**_*q*_‖ < ‖*δ***x**_*q*−1_‖      $\mu =\frac{\mu }{\lambda };$      else      *μ* = *μλ*.      end if     end if   end for
**Output:**
**x**_*q*_


### 3.3. Computational Complexity

We estimate the computational complexity of the proposed positioning algorithm in this subsection. The complexity is shown in big *O* expressions in terms of the number of anchors *M*, the number of antennas *N*, and the position dimension 2. 

To investigate the worst case, it is assumed that all *N* antennas can each receive all *M* anchor signals, although this situation is unlikely to occur due to the obstacles. Step 1 has MN+C(N,2) measurements and 2N+1 unknown states. In each iteration of the procedure to estimate the antennas positions, the complexities to calculate δq, K, and ${\left(K^{T}WK\right)}^{-1}$ are O(MN+C(N,2)), O((2N+1)(MN+C(N,2))), and $O\left(\left(2N+1\right){\left(MN+C\left(N,2\right)\right)}^{2}\right)$ flops, respectively. Moreover, the procedure to obtain the coarse estimate takes $O(2^{3})$ flops for an SVD. Step 2 has MN measurements and four unknown states. In each iteration of the procedure to estimate the refined solutions, the complexities to calculate δz,H, and ${Q^{\prime }}^{-1}$ are O(MN), O(4MN), and $O\left(4{\left(MN\right)}^{2}\right)$ flops, respectively.

The involved operations are matrix inversion, SVD, and other ordinary addition and multiplication steps. Although the algorithm is implemented on a personal computer (PC) for our simulation tests in Section 5, it is expected that the proposed method can be realized with reasonable computational cost in embedded systems commonly adopted by AGVs.

## 4. Performance Metrics

In this section, availability and accuracy are referred to as the performance metrics for the issue introduced above. The availability is the time percentage through which the positioning service is available, taking into consideration the needed accuracy and integrity [13]. In this paper, it is simplified to the solvability of the positioning parameter estimation, which depends on whether the number of effective independent measurements is greater than the minimum number of measurements required and the geometry of the visible anchors. In the DMA method, the availability is expressed as the ratio of time satisfying Equation (10) to the total time. As for the accuracy, CRLB and the dilution of precision (DOP) are usually used as a performance measure. Although there are mature theoretical models of CRLB and DOP for conventional single-antenna positioning, we need to specifically derive the models for the DMA method.

### 4.1. CRLB

To evaluate the estimator, we derive the CRLB for the DMA method, which is the lower bound of the error variance of the unbiased estimator. The error variance of x[l] is as follows [44]:

(47) $$\mathrm{var}\left(\hat{x}\left[l\right]\right)\geq CRLB\left(\hat{x}\left[l\right]\right)={\left[{\mathrm{FIM}}^{-1}(x)\right]}_{l,l},$$

where FIM is the Fisher information matrix.

The entries of the Fisher information matrix are

(48) $${\left[\mathrm{FIM}(x)\right]}_{l_{1},l_{2}}=-E\left[\frac{\partial^{2}\ln p(z;x)}{\partial x\left[l_{1}\right]\partial x\left[l_{2}\right]}\right],$$

where p(z;x) is the likelihood function.

We can define $g_{n}(x)$ as the *n*-th residual of Equation (7), where $n=1,2,\cdots ,\sum_{i=1}^{N}M_{i}$. Thus, $g_{n}(x)∼N\left(0,\sigma^{2}\right)$. The probability density function of each TOA measurement z[n] can be expressed as

(49) $$p(z[n];x)=\frac{1}{\sqrt{2\pi \sigma^{2}}}\exp\left(-\frac{1}{2\sigma^{2}}g_{n}{\left(x\right)}^{2}\right).$$

Each measurement is measured independently; thus,

(50) $$p(z;x)=\prod_{n=1}^{L}p(z\left[n\right];x)=\frac{1}{{\left(2\pi \sigma^{2}\right)}^{\frac{L}{2}}}\exp\left(-\frac{1}{2\sigma^{2}}\sum_{n=1}^{L}{\left(g_{n}(x)\right)}^{2}\right),$$

where $L=\sum_{i=1}^{N}M_{i}$.

Therefore, the Fisher information matrix FIM is

(51) $$\mathrm{FIM}=-E\left[\frac{\partial^{2}\ln p(z;x)}{\partial x\partial x^{Τ}}\right]=\frac{1}{\sigma^{2}}\sum_{n=1}^{L}{\left(\frac{\partial g_{n}(x)}{\partial x}\right)}^{T}\frac{\partial g_{n}(x)}{\partial x}=\frac{1}{\sigma^{2}}H^{T}H,$$

where H is defined in Equation (42). 

Consequently, the diagonal element of ${\mathrm{FIM}}^{-1}$ is the minimum variance that can be achieved theoretically in unbiased estimation for the DMA positioning method. To simplify and save space, the noise of each measurement is set to be independent and identically distributed. If the signal transmitted from an anchor or received by an antenna is interfered, it can be considered that the corresponding noise increases, resulting in a decrease in precision. Furthermore, the multipath has a complicated effect on TOA measurements. With respect to CRLB, the multipath equivalently increases the measurement noise, and then reduces the positioning accuracy. As there is a certain amount of work in multipath modeling, it will be further studied in future work.

Note that, in Equation (51), FIM is determined by H with the same measurement noise. Moreover, H is affected by the relative position between antennas and their visible anchors. Thus, the position and quantity of antennas will affect positioning accuracy. However, we mainly focus on providing a solution for AGV positioning with severe blockages in this work. The quantitative influence of antenna configuration and the criteria of antenna position and quantity configuration will be studied in future work.

Based on the theoretical CRLB, the positioning performance of the proposed method is tested using a simulation in Section 5.

### 4.2. DMA-HDOP 

In this subsection, DOP is introduced to analyze the positioning accuracy. Considering the measurement noises ε and adding the estimate error $\varepsilon_{x}$ caused by ε, Equation (40) can be written as follows [45]:

(52) $$\delta z=H\left(\delta x+\varepsilon_{x}\right)+\varepsilon .$$

Then,

(53) $$\delta x+\varepsilon_{x}={\left(H^{T}H\right)}^{-1}H^{T}\delta z-{\left(H^{T}H\right)}^{-1}H^{T}\varepsilon .$$

According to Equation (45), we have

(54) $$\varepsilon_{x}=-{\left(H^{T}H\right)}^{-1}H^{T}\varepsilon .$$

The covariance matrix of positioning error is

(55) $$\begin{array}{ll} \mathrm{cov}\left(\varepsilon_{x}\right) & =E\left[\varepsilon_{x}\varepsilon_{x}^{T}\right]\\ & =E\left[-{\left(H^{T}H\right)}^{-1}H^{T}\varepsilon {\left(-{\left(H^{T}H\right)}^{-1}H^{T}\varepsilon \right)}^{T}\right]\\ & ={\left(H^{T}H\right)}^{-1}\mathrm{cov}\left(\varepsilon \right) \end{array}$$

According to the discussion of the TOA measurements noise in Section 2.3, the covariance matrix of ε is $\mathrm{cov}\left(\varepsilon \right)=\sigma^{2}I$. Thus,

(56) $$\mathrm{cov}\left(\varepsilon_{x}\right)={\left(H^{T}H\right)}^{-1}\sigma^{2}.$$

This can be denoted as

(57) $$G={\left(H^{T}H\right)}^{-1}=\left[\begin{array}{cccc} g_{11} & g_{12} & g_{13} & g_{14}\\ g_{21} & g_{22} & g_{23} & g_{24}\\ g_{31} & g_{32} & g_{33} & g_{34}\\ g_{41} & g_{42} & g_{43} & g_{44} \end{array}\right].$$

The diagonal elements of G are the variance of state estimation errors, while the non-diagonal elements represent the correlation between these states. G indicates the magnification of the measurement error. With the same measurement error, a greater G results in a lower positioning accuracy. The DMA-HDOP can be expressed as

(58) $$\mathrm{DMA}\text{-}\mathrm{HDOP}=\sqrt{g_{11}+g_{22}}.$$

In Section 5, the positioning accuracy is analyzed based on the DMA-HDOP model.

## 5. Simulation Results

**Figure 5:**
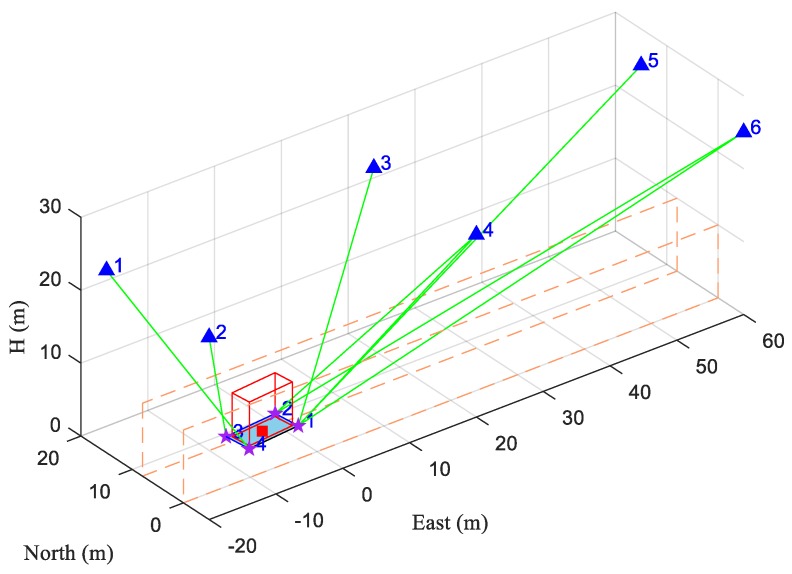
Simulation scene. The blue triangles numbered from 1–6 represent the anchors, the purple stars numbered from 1–4 indicate the antennas mounted on the AGV, the red solid square indicates the center of the AGV (reference point), the red hollow cube in the center indicates the cargo on the AGV, the rectangle filled with blue indicates the AGV body, the rectangles with orange dashed lines indicate the walls on both sides of the road, and the green solid lines indicate the lines of sight (LOSs) from the antennas to their visible anchors.

To analyze the performance of the proposed method, several tests were conducted based on one simulation scene. As shown in Figure 5, we set up a scene with six anchors, an AGV, and two wall-like obstacles along both sides in the environment. There is some cargo on the flat of the AGV. Its length and width are smaller than that of the flat, which means that its dimensions are not outside the AGV. Both the walls and the cargo on board can block the positioning signals. Four strictly synchronized receiving antennas are mounted on the corners of the AGV. We set the center of the AGV as the reference point.

For this scene, M=6, N=4. The height of both walls was 10 m, the width of the road between the walls was 8 m, and the coordinates of four antennas in frame *b* $l_{ci}^{b}$ were (4, 2, 0) m, (4, −2, 0) m, (−4, −2, 0) m and (−4, 2, 0) m, respectively. The coordinates of the six anchors in frame *n* are presented in Table 1. The length, width, and height of the cargo on the vehicle were 7 m, 3 m, and 6 m, respectively. We ignored the multipath effect here and the TOA measurement noise was considered as independent zero-mean Gaussian white noise with a standard deviation of σ=0.2 m. The clock bias between the receiver and the anchors was 149.90 m. The visible anchor set of each antenna and the corresponding TOA measurements from each antenna were then generated at a 1-Hz update rate.

Our simulation was carried out in MATLAB R2017a on a PC with an Intel(R) core(TM) i7-6500U central processing unit (CPU) at 2.5 GHz. By using the simulated measurements, we examine the theoretical capability of the proposed method in terms of availability and accuracy in Section 5.1. In Section 5.2, the performance of the TSLM algorithm and the influence of the initial value on the iterative positioning algorithm are tested.

### 5.1. Theoretical Capability of DMA Method

**Figure 6:**
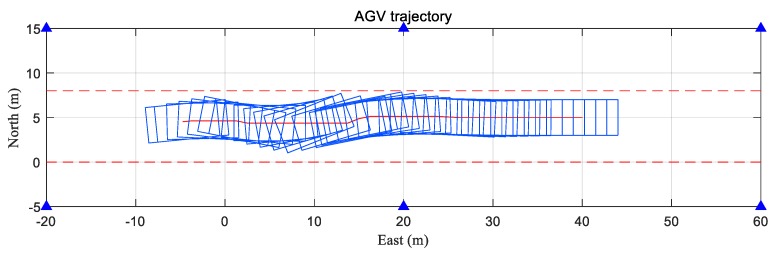
The trajectory of the center and the attitude of the AGV in the dynamic test. The blue triangles represent the anchors, the red line indicates the trajectory of the center, the blue rectangle indicates the AGV body, and the red dashed lines indicate obstacles on both sides of the road.

Based on the above configuration, we carried out a dynamic simulation test to assess the theoretical capability of the DMA method. The trajectory of the center and the attitude of the AGV were plotted in a 2D projection, as shown in Figure 6. The vehicle moved along the road from west to east with varying yaw angle and north velocity. The vehicle started from (−4.75, 4.53, 2.4) m with an initial attitude angle of (0, 0, 1.47) rad. During the movement, the height, rolling angle, and pitch angle of the vehicle remained unchanged. The duration of the movement was 180 s, which was divided into 11 segments according to different motion states. With different position and attitude of the AGV, each moment of simulation had a different geometric distribution of the anchors and actually represented many different situations. The east velocity $v_{E}$, north velocity $v_{N}$, yaw angle rate ω, and duration T of each segment are shown in Table 2. Utilizing the above scene and parameters, the visible anchor set of each antenna and the corresponding TOA measurements from each antenna of every moment were generated.

#### 5.1.1. Positioning Availability

**Figure 7:**
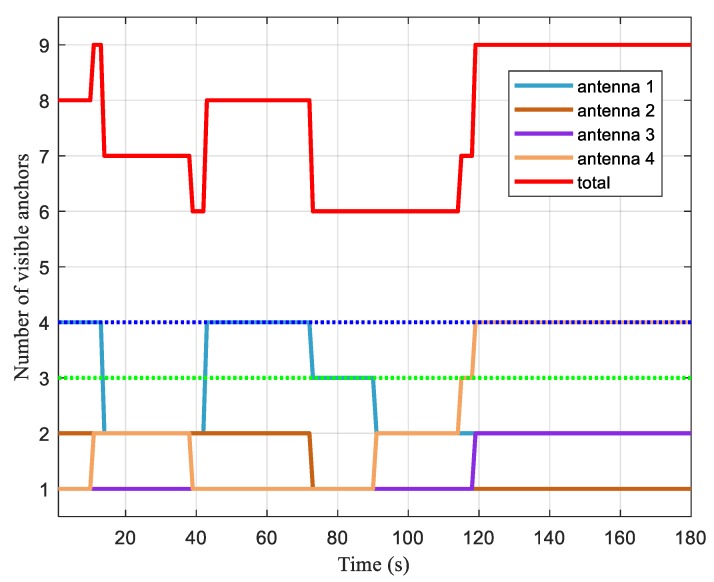
The numbers of visible anchors vs. time.

As mentioned in Section 4, we analyzed the availability by the ratio of time satisfying Equation (10) to the total time. Due to the movement of the vehicle, the numbers of visible anchors of each antenna changed during the test, as shown in Figure 7. 

In Figure 7, the minimum numbers of visible anchors required for the single-antenna method and DMA positioning method are also plotted as the green dotted line and blue dotted line, respectively. The lines denoting the visible anchor numbers of antenna 2 and antenna 3 were always below the green dotted line, while antenna 1 and antenna 4 had more than three visible anchors at some moments. That is to say, single-antenna positioning was not available during parts of this test. On the other hand, the line of the total number of visible anchors was always above the blue dotted line. This indicates a possible positioning for the AGV if all measurements were used. Furthermore, there were always more than two antennas with one or more visible anchors, indicating that the yaw angle could be determined. Therefore, the condition in Equation (10) was satisfied throughout the test. We combined the numbers of visible anchors of antennas 1 and 4, and calculated the proportion of time when the number of visible anchors exceeded three (the green dotted line in Figure 7). The result was 70.6%, which means that the single-antenna method was only available during 70.6% of the entire period. With respect to the DMA method, the availability increased to 100% because the total number of visible anchors was above four (the blue dotted line in Figure 7) throughout the simulation period. This verifies that, with obstacles both in the environment and on the AGV, the proposed DMA method significantly improved the positioning availability in this simulation.

#### 5.1.2. Positioning Accuracy

In this section, we analyze the positioning accuracy using DMA-HDOP and the theoretical CRLB derived in Section 4. 

**Figure 8:**
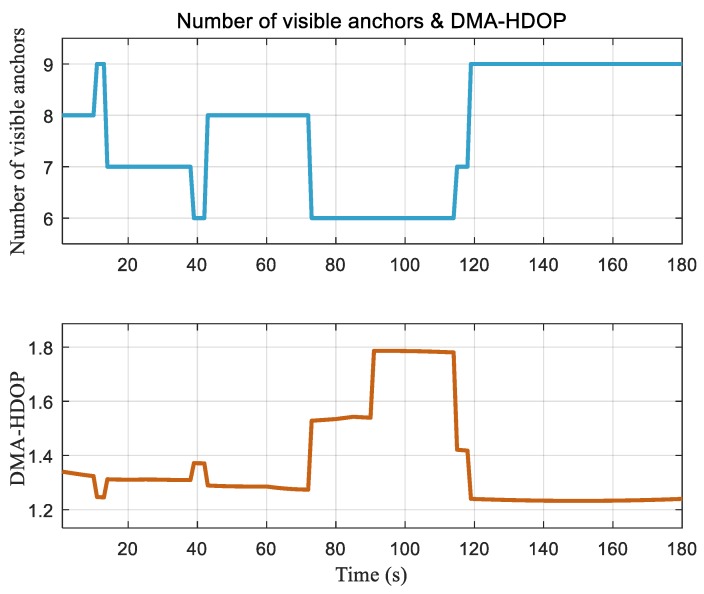
Number of visible anchors of multiple antennas (top) and distributed multi-antenna horizontal dilution of precision (DMA-HDOP) (bottom).

**Figure 9:**
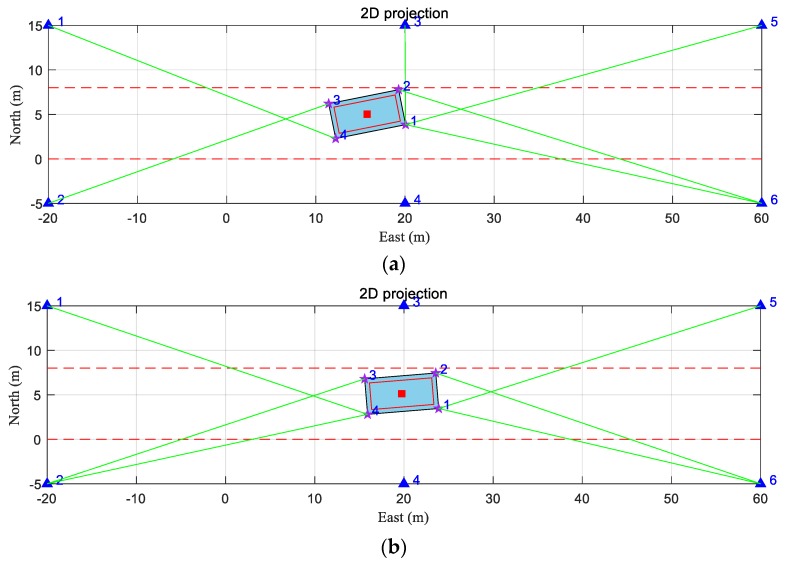
Two-dimensional (2D) projection at (**a**) 84 s, and (**b**) 100 s. Blue triangles numbered from 1–6 indicate anchors, purple stars numbered from 1–4 indicate antennas mounted on the corner of the AGV, the red solid square indicates the center of the AGV, the red rectangle indicates the projection of the cargo on AGV, the rectangle filled with blue indicates the AGV body, the red dashed lines indicate the projections of the walls on both sides of the road, and green lines indicate the LOSs from the antennas to their visible anchors.

Based on the above scene and parameter configuration, the DMA-HDOP was calculated according to Equation (58), as shown in Figure 8. The total number of visible anchors is also shown for comparison. It can be seen from Figure 8 that the change in DMA-HDOP value was consistent with that of the number of visible anchors. A greater number of visible anchors led to a smaller DMA-HDOP value; thus, with the same measurement error, we obtained a smaller positioning error. Note that the number of visible anchors did not change between 74 s and 114 s, while the DMA-HDOP value did. This is because, during this period, the geometry of visible anchors changed when the AGV moved. Taking 84 s and 100 s as examples, as shown in Figure 9, although the numbers of visible anchors were the same, the geometric distribution was more uniform at 84 s due to the reception by the distributed multi-antenna.

**Figure 10:**
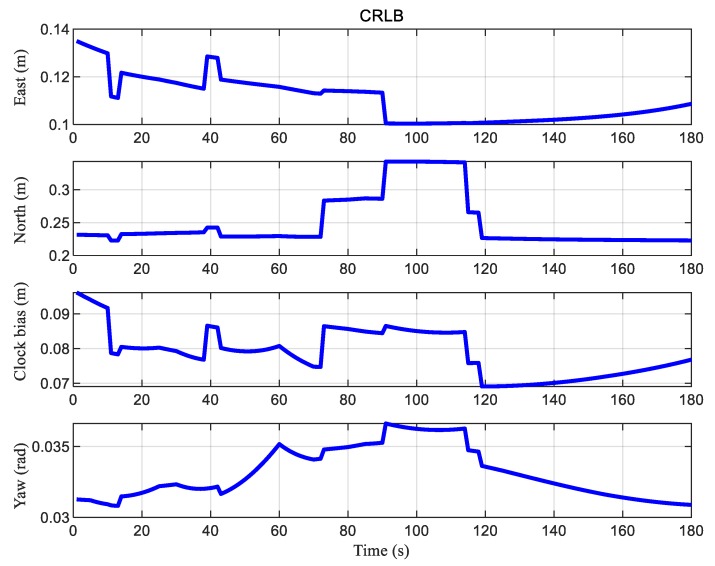
The Cramer–Rao lower bound (CRLB) of DMA positioning at each moment.

Next, the simulated measurements were used to calculate the theoretical CRLB according to Equation (47). As shown in Figure 10, the CRLB of DMA positioning was also consistent with the numbers of visible anchors at each moment. In addition, in line with the analysis of Figure 9, the error in the north direction was larger between 90 s and 114 s. Overall, according to CRLB, with the standard deviation of the TOA measurement noise as σ=0.2 m, the DMA method could theoretically achieve decimeter-level accuracy.

### 5.2. Performance of TSLM Algorithm

**Figure 11:**
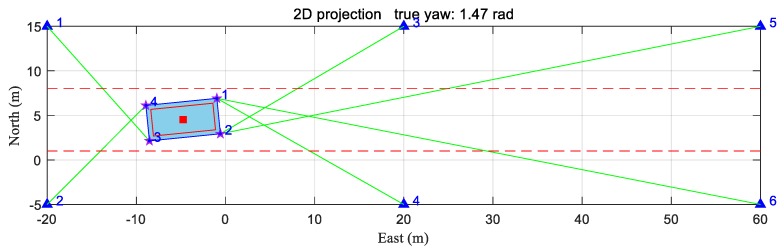
The 2D projection at the start point of the trajectory.

In this subsection, we conducted a static test at the start point of the trajectory in Figure 7 to test the performance of TSLM algorithm and the influence of the initial yaw angle on the iterative algorithm. In this test, the true coordinates of the vehicle were (−4.75, 4.53, 2.4) m with the true attitude angles as (0, 0, 1.47) rad. The 2D projection is shown in Figure 11, with the same icons and symbols as those in Figure 9. 

Because of the blockage by the obstacles both in the environment and on the AGV, the numbers of signals received by the antennas on the vehicle were two, two, one, and one. None of the antennas had more than three visible anchors; thus, none of them could complete positioning on their own. However, the total number of visible anchors was greater than four and all four antennas had their visible anchor. Equation (10) was satisfied; therefore, the proposed positioning algorithm could be applied. Furthermore, the total number of visible anchors satisfied Equation (17); thus, the coarse estimation process could be applied as well. 

To compare its positioning performance with CRLB, the positioning errors were calculated by comparing the simulation results with the true values of the simulation setting. A Monte Carlo simulation over 1000 tests was implemented to obtain the root-mean-square error (RMSE). For each parameter ξ, the RMSE can be calculated by

(59) $$\mathrm{RMSE}=\sqrt{\frac{\sum_{i=1}^{N_{\mathrm{test}}}{\left(\xi -{\hat{\xi }}_{i}\right)}^{2}}{N_{\mathrm{test}}}},$$

where ${\hat{\xi }}_{i}$ is the estimation of ξ in test *i*, $N_{\mathrm{test}}$ is the number of Monte Carlo simulation tests, and $N_{\mathrm{test}}=1000$ in this simulation. 

**Figure 12:**
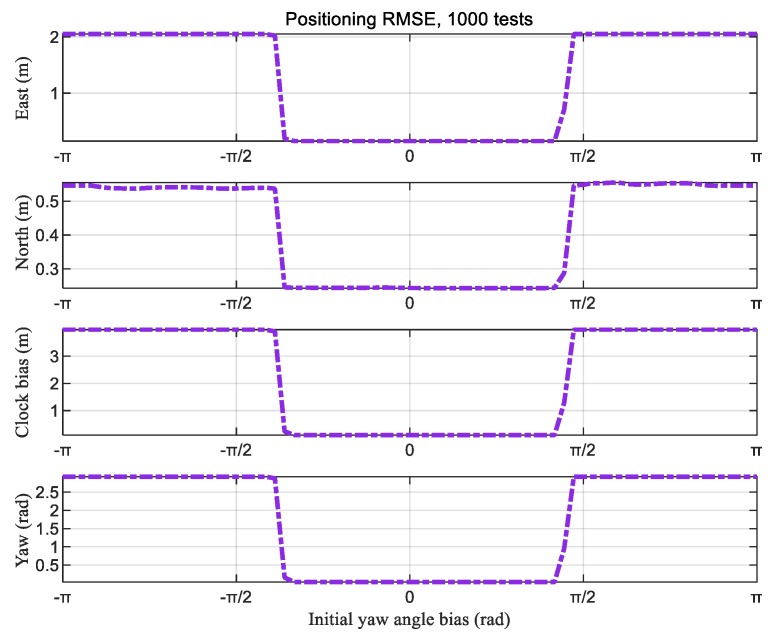
Root-mean-square errors (RMSEs) of Step 2 vs. initial yaw angle bias.

In order to evaluate the impact of initial yaw angle and validate the necessity and effectiveness of Step 1 in the proposed algorithm, we firstly imported the simulated measurements directly to Step 2, i.e., the iterative positioning algorithm. For Step 2, $\delta t_{0}$ was set to 0, and $\left(x_{c|0},y_{c|0}\right)$ was set as the center of the range formed by the anchors. With the initial yaw angle bias varying from −*π* rad to *π* rad, the positioning results were calculated. By substituting simulation results into Equation (59), the positioning RMSEs of Step 2 could be determined, as presented in Figure 12. The positioning RMSEs fluctuated severely with the initial yaw angle bias, which indicates that the iterative positioning algorithm was sensitive to the initial yaw angle and was consistent with the analysis in the Section 3.

The simulated measurements were then imported into TSLM, with $\delta t_{0}=0\;m$, and initial positions of the distributed antennas as four random points near the center of the area formed by the anchors. The RMSE of the yaw angle obtained in Step 1 was 0.1829 rad, which means that the coarse solution was not far from the optimum. By using coarsely estimated solutions of Step 1, the refined solutions were obtained. 

Utilizing the above simulation results, CRLB was computed based on Equation (44) using the true positions of AGV and anchors in the simulation settings. The positioning RMSEs of the proposed algorithm from 1000 Monte Carlo simulation tests were computed based on Equation (59). The positioning RMSEs of our algorithm and the theoretical CRLB of the DMA method are presented in Table 3. In this table, the results of the “only Step 2” column in the table were calculated with different representative initial values of $\psi_{c|0}$ as −1.67 rad, 3.04 rad, and 1.79 rad, representing −π rad, π/2 rad, and π/10 rad bias with respect to the true yaw angle, respectively. When the bias was π/10 rad, the positioning RMSEs were also close to the theoretical CRLB, as with those of the TSLM algorithm. However, when the bias was −π rad or π/2 rad, the positioning RMSEs from Step 2 only were far greater than for CRLB. This result indicates that, when the initial value is far away from the true value, the algorithm may converge to the local optimal solution, presenting the wrong position.

The simulation results verify that, without the prior information of the yaw angle, the TSLM algorithm avoids the initial yaw angle problem, and it can obtain a positioning accuracy close to the theoretical lower bound by using coarse estimation. Furthermore, if there is prior attitude information of the body, using Step 2 only can also achieve the desired accuracy.

## 6. Conclusions

Autonomous high-precision positioning and navigation of AGV are desperately needed in unmanned warehouses, ports, and similar environments. Radio broadcast positioning systems can be employed in these AGV positioning applications. Due to the limitation of the flat structure of AGV, the receiving antennas cannot be mounted at the highest point of the vehicle. The positioning signals are not only affected by the obstacles from the environment but also blocked by the AGV cargo. The availability and accuracy of the positioning system are severely challenged. 

To tackle the above problem, a distributed multi-antenna positioning method was proposed, which utilizes the synchronous reception by multiple antennas. In this method, the measurements of the distributed multiple antennas are transformed to a function of the reference position by estimating the attitude of the body. To estimate the position, receiver clock bias, and attitude angle, a two-step positioning algorithm TSLM was proposed. Firstly, the positions of the distributed antennas and the clock bias are jointly estimated. Then, the reference position and the yaw angle are estimated according to the transformation of antenna positions between frame *b* and frame *n*. Through Step 1, the unknowns are coarsely estimated to a proper accuracy, so that they can be applied as initial values for Step 2 to guarantee its convergence to the global optimum. Finally, a refined positioning result can be obtained iteratively in Step 2. Simulation results demonstrated that, compared with the conventional single-antenna localization, the positioning availability was significantly improved. Moreover, with the standard deviation of the TOA measurement noise as 0.2 m, the DMA method could achieve decimeter-level accuracy. Simulation tests verified that the TSLM algorithm can provide a position estimation with an accuracy close to CRLB without the requirement for initial attitude information. Furthermore, with prior information of the yaw angle, Step 2 can achieve similar results to TSLM. 

In the future, more factors such as multipath and non-line-of-sight (NLOS) will be considered in algorithm design and performance analysis, and more studies will be done on measurement noise modeling. In addition, the theoretical analysis of the influence of the initial values and the effect of the proposed algorithm will be carried out. The nonlinear constrained optimization problem will be further studied to extend the proposed method to 3D positioning and attitude determination. Improvements on computational complexity and other algorithms with possibly higher efficiency and better robustness will be investigated. Moreover, the quantitative influence of antenna configuration and the criteria of antenna position and quantity configuration will also be studied.

## Figures and Tables

**Table 1:** The coordinates of the six anchors in frame *n* (m).

Anchor No.	1	2	3	4	5	6
East	−20	−20	20	20	60	60
North	15	−5	15	−5	15	−5
Up	25	25	25	25	25	25

**Table 2:** The parameters of the AGV movement.

Segment	1	2	3	4	5	6	7	8	9	10	11
$v_{E}$ (m/s)	0.25	0.25	0.25	0.25	0.375	0.125	0.25	0.25	0.25	0.25	0.25
$v_{N}$ (m/s)	0.025	0	−0.05	0	0.05	0.05	0.05	0	−0.013	0	0
ω (rad/s)	0	−0.015	0	0.019	−0.018	0	0	−0.008	0	0.002	0
T (s)	5	20	5	30	10	5	10	30	10	30	25

**Table 3:** The positioning RMSEs of the proposed algorithm and the CRLB of the DMA method. This table lists the positioning RMSEs of the proposed TSLM algorithm and Step 2 only of the TSLM algorithm. The results of Step 2 only are given with different initial yaw angles. CRLB is listed as a benchmark.

Parameters	RMSE				CRLB
	TSLM	Only Step 2			
		bias=−π rad	bias=π/2 rad	bias=π/10 rad	
*x* (m)	0.146	2.053	2.053	0.146	0.144
*y* (m)	0.244	0.546	0.549	0.243	0.242
Clock bias (m)	0.099	3.980	3.981	0.099	0.099
Yaw angle (rad)	0.033	2.924	2.924	0.033	0.032
